# Metabolic Markers of MRI-Confirmed Lacunar Stroke: Observational and Mendelian Randomization Analyses

**DOI:** 10.1212/WNL.0000000000218409

**Published:** 2026-09-10

**Authors:** Wen Hui Ng, Elnara Aghakishiyeva, Hugh S. Markus, Eric L. Harshfield

**Affiliations:** From the Stroke Research Group, Department of Clinical Neurosciences, University of Cambridge, United Kingdom.

## Abstract

**Background and Objectives:**

Lacunar stroke results from cerebral small vessel disease (SVD) and is a major cause of vascular dementia and cognitive decline. We aimed to evaluate the associations of metabolites with lacunar stroke, neuroimaging markers of SVD, and cognition to help elucidate SVD pathogenesis.

**Methods:**

In a clinical cohort of MRI-confirmed lacunar stroke cases and unrelated hospital controls recruited from stroke centers across the United Kingdom, we assayed 250 serum metabolites using NMR spectroscopy. We investigated the association of metabolites with MRI-confirmed lacunar stroke, neuroimaging markers, and cognitive impairment. We also conducted a genome-wide association analysis for each metabolite and applied bidirectional Mendelian randomization (MR) to investigate causality.

**Results:**

Among 2,408 participants, there were 1,456 lacunar stroke cases (mean age, 62 [SD 12]; 34% female) and 952 controls (mean age, 57 [SD 6]; 38% female). Observational analyses identified 211 metabolites that were significantly associated with lacunar stroke. MR analyses identified significant evidence to support causal associations of 2 of these metabolites, glycine and the proportion of cholesterol to total lipids within medium low-density lipoprotein (LDL) (%C/Total in M-LDL), with 2 distinct lacunar stroke subtypes—isolated lacunar infarcts (ILI, 1 lacunar infarct with Fazekas grade <2) and multiple lacunar infarcts and/or leukoaraiosis (MLI/LA, >1 lacunar infarct and/or Fazekas grade ≥2). Glycine was inversely associated with ILI (OR: 0.704, 95% CI 0.585–0.848), and %C/Total in M-LDL was inversely associated with MLI/LA (OR: 0.541, 95% CI 0.401–0.730). Five metabolites (Total S-high-density lipoprotein [HDL], S-HDL concentration, phospholipid (PL) in S-HDL, %CE/Total in L-LDL, and lactate) were significantly associated with executive functioning and processing speed in observational analyses. MR analyses also implicated genetically determined levels of ω-3/Total FA with 2 diffusion tensor imaging markers of SVD. Overall, the most robust and consistent associations across both observational and MR analyses were identified for glycine and %C/Total in M-LDL with lacunar stroke subtypes.

**Discussion:**

We identified inverse associations of glycine and the proportion of cholesterol to total lipids within medium LDL with distinct subtypes of MRI-confirmed lacunar stroke. These findings reveal metabolomics signatures associated with lacunar stroke subtypes, which may inform future studies of SVD pathogenesis and biomarker development.

## Introduction

Cerebral small vessel disease (SVD) affects the small perforating arteries, arterioles, capillaries, and small veins in the brain^1^; causes lacunar stroke and most deep intracerebral hemorrhages; and is the leading pathology underlying vascular dementia.^2-4^ Characteristic MRI features include lacunar infarcts, cerebral microbleeds, and white matter hyperintensities (WMHs).^5^

SVD can be broadly categorized into amyloid-related (primarily cerebral amyloid angiopathy) and non–amyloid-related forms, which include sporadic subcortical SVD (commonly associated with hypertension) and hereditary conditions (e.g., cerebral autosomal dominant arteriopathy with subcortical infarcts and leukoencephalopathy).^6^ In sporadic SVD, the primary pathology affects the small perforating end-arteries, which can be affected by intrinsic pathologic changes, such as lipohyalinosis (diffuse abnormality of the small vessels), or have their flow reduced by atherosclerotic plaques at their origins.^6^ Pathologic and radiologic studies have suggested 2 sporadic SVD types, with distinct vascular risk profiles: (1) single, larger lacunar infarcts due to microatheroma and (2) multiple, smaller lacunar infarcts resulting from lipohyalinosis, often associated with confluent WMH (leukoaraiosis).^7-9^ Lacunes typically occur near and are linked mechanistically to preexisting WMH.^10^

Despite its clinical significance, the pathogenesis of SVD remains poorly understood.^11^ Consequently, current treatment and prevention strategies for SVD are largely restricted to managing established vascular risk factors.^2^ Improving understanding of SVD pathophysiology and identifying novel risk factors are critical steps toward developing targeted interventions to prevent stroke and reduce cognitive decline.

Metabolomics profiling provides a powerful tool for identifying novel risk factors in SVD by enabling comprehensive characterization of metabolic pathways implicated in disease pathogenesis and potentially enhancing prediction of disease progression and severity.^12^ High-throughput techniques (e.g., NMR spectroscopy) allow quantification of a broad spectrum of metabolites with high sensitivity and specificity.^13^ Although metabolomics has been widely applied to cardiovascular and neurologic diseases,^14,15^ relatively few studies have investigated it in SVD. Previous studies reported associations between metabolites and neuroimaging features of SVD in community-based populations^16^ and smaller cohorts,^12,17^ but there remain few studies in large cohorts of lacunar stroke patients, and limited data on metabolite associations with vascular cognitive impairment (VCI).

In this study, we compared metabolite concentrations between well-phenotyped, MRI-confirmed lacunar stroke patients and stroke-free controls using data from 2 longitudinal, prospectively recruited, UK-based, multicenter studies. We examined associations of metabolites with lacunar stroke subtypes, neuroimaging markers of SVD, and cognitive function. By integrating evidence from observational and causal inference analyses, we aimed to identify novel and robust metabolic biomarkers for lacunar stroke.

## Methods

The study is reported following STROBE (Strengthening the Reporting of Observational Studies in Epidemiology) guidelines for observational studies.

### Study Population

DNA Lacunar 1 (DL1) and 2 (DL2) are prospective studies of MRI-confirmed lacunar stroke patients recruited from UK stroke centers. DL1 recruited 1,030 lacunar stroke patients of European ancestry aged 70 years or older from 72 specialist stroke centers between 2002 and 2012.^18^ DL1 also recruited 1,973 unrelated hospital controls of European ancestry without a history of stroke, myocardial infarction, or dementia, who were randomly recruited from general practices covering the same geographical area as the cases.^18^ DL2 had recruited 1,319 MRI-confirmed lacunar stroke patients from 54 hospitals as of July 2023^19^ (recruitment is ongoing). Further details are in the eMethods.

### Review of MRI

MRI scans, performed clinically at each recruiting site, were reviewed centrally to confirm eligibility. MRI included diffusion-weighted imaging (DWI) sequences to identify acute infarcts, fluid-attenuated inversion recovery (FLAIR) and/or T1 sequences to count old lacunar infarcts, and FLAIR and/or T2 sequences to identify WMH.^20^ Susceptibility-weighted imaging and gradient echo sequences, if available, were used to count cerebral microbleeds. Lacunar stroke was defined by the presence of a clinical lacunar syndrome with an anatomically compatible lesion on MRI (DWI-positive lesion for acute infarcts or a round/oval subcortical fluid-filled cavity 3–15 mm in diameter for cases imaged >3 weeks after the acute event).^19^ Microbleeds were defined as focal areas of very low intensity <10 mm in diameter and counted using the Brain Observer Microbleed Rating Scale.^21^ WMHs were graded using the Fazekas scale by 3 trained neurologists. The Cohen kappa interrater reliability test showed good-to-very-substantial agreement (*κ* = 0.788).^20^ Lacunar stroke cases were classified into subtypes based on MRI scans. Isolated lacunar infarcts (ILI) and multiple lacunar infarcts (MLI) were defined as 1 or >1 lacunar infarct, respectively, without confluent WMH (Fazekas grade <2). Leukoaraiosis (LA) was defined as having confluent WMH (Fazekas grade ≥2) irrespective of the number of lacunar infarcts. MLI and LA were combined, consistent with previous studies.^22^

### Cognitive Assessment

DL2 cases were assessed using the Brief Memory and Executive Test (BMET),^20,23^ a short cognitive screening tool designed and validated to be sensitive for detection of VCI in SVD.^23^ Cognitive assessment took place on the day of consent, a median of 25 days (interquartile range 4–105 days) after stroke onset, so most participants were assessed during the acute or subacute phases. The BMET includes a number of subscores, each ranked on an age-normative scale from 0 to 2, which are used to form a total score ranging from 0 to 16.^23^ A BMET total score ≤13 has been validated to indicate the presence of VCI.^23^ Test performance was subdivided into 2 major cognitive domain clusters—orientation/memory (O/M) and executive functioning/processing speed (EF/PS)—each with a score ranging from 0 to 8. The O/M subdomain score was calculated from the orientation, 5-item repetition, 5-item recall, and 5-item recognition memory tasks, while the EF/PS subdomain score was calculated from the letter-number matching, motor sequencing, letter sequencing, and number-letter sequencing tasks.^23^

### Metabolomics Profiling

We followed a structured, criterion-based selection process to select serum samples from 2,500 participants (673 DL1 cases, 827 DL2 cases, and 1,000 DL1 controls) for metabolomics profiling. All selected cases had lacunar stroke confirmed on central review of imaging. Following selection, samples in 0.5 mL tubes in −80°C freezers were transferred to twenty-seven 96-well microtube racks. We distributed tubes to prevent study or disease-status batch effects, ensuring that each rack contained up to 25 DL1 cases, 31 DL2 cases, and 38 DL1 controls, with at least 2 empty wells for QC samples.

Metabolic biomarker profiling was performed at Nightingale Health's laboratory in Finland in September 2023. Serum samples were shipped on dry ice. High-throughput NMR spectroscopy was used to obtain measurements of 250 metabolites for each sample with high precision and accuracy (median intraclass coefficient: 0.99).^13,24^ All samples were processed on a single spectrometer. Advanced proprietary algorithms translated the NMR spectral data into absolute quantifications of metabolites.^13^ The platform measured diverse biomarkers including amino acids, apolipoproteins, cholesterol and cholesteryl esters, fatty acids, phospholipids, triglycerides, and detailed lipid concentrations and compositions within 14 different lipoprotein subclasses (eTable 1).^13^

### Statistical Analyses

#### Observational Analyses

To ensure approximately normal distributions, a generalized log transformation was applied to each metabolite, and concentrations were rescaled using mean centering and dividing by the SD of each metabolite across participants. Batch correction was not necessary because of high repeatability and negligible batch effects and because all samples were analyzed on a single mass spectrometer.^13,24^ We excluded samples with insufficient sample volume (minimum 350 µL) and measurements below the limit of quantification.

The presence of confluent WMH was defined as Fazekas grade ≥2, and VCI was defined as BMET total score ≤13. Age-normalized scores for the BMET O/M and EF/PS subdomains were analyzed as continuous outcomes—the directions were reversed so that higher scores indicated evidence of cognitive impairment.

In our primary analyses, we studied the association of metabolites with lacunar stroke cases vs controls, and with ILI and MLI/LA subtypes vs controls, using logistic regression models adjusted for age and sex. Within lacunar stroke cases (i.e., excluding controls), we also analyzed the association of each metabolite with the presence of WMH, cerebral microbleeds, and VCI using logistic regression, and with the BMET O/M and EF/PS subdomain scores using linear regression, all adjusted for age and sex.

As sensitivity analyses, we conducted regression analyses for the association of each metabolite with the above outcomes with further adjustment for hypertension, smoking, alcohol consumption, body mass index (BMI), hyperlipidemia, and type 2 diabetes. We also conducted analyses with further adjustment for medications commonly prescribed for stroke treatment—including antihypertensives, diabetic drugs, lipid-lowering drugs, antiplatelets, and anticoagulants—that patients were taking before the stroke event and/or recruitment. Furthermore, we conducted analyses within cases adjusted for the number of days between the stroke event and the date of blood sample collection.

We also performed a principal component analysis (PCA) of metabolite levels and assessed the association of the top principal components (PC), which explained the greatest proportion of the variance in metabolite concentrations, with each of the above outcomes with adjustment for age and sex.

Observational analyses were conducted in R v4.4.1. A false discovery rate (FDR) threshold of 5% was used to account for testing associations with 250 metabolites for each outcome.

#### Causal Inference Analyses

To complement the observational analyses, we conducted a genome-wide association analysis for each of the 250 metabolites using genetic data from DL1 and DL2, and obtained genetic association summary statistics for MRI-confirmed lacunar stroke, ILI, MLI/LA, and neuroimaging markers from published studies.^25,26^ See the eMethods for further details of genotyping, imputation, quality control, genome-wide association analyses, and genetic association summary statistics. We performed 2-sample Mendelian randomization analyses (MR) using the single-nucleotide polymorphisms independently associated with each metabolite as instrumental variables to examine evidence for a causal relationship of metabolites with lacunar stroke and MRI markers of SVD. Primary analyses were conducted using the inverse-variance–weighted (IVW) method with an LD clumping threshold of *r*^2^ < 0.01.

Sensitivity analyses were performed including the weighted median, simple and weighted mode, and MR-Egger regression methods, which require different assumptions to be satisfied, thus supporting the credibility of inferred causal claims.^27^ Cochran Q-statistic from the IVW method was used to diagnose heterogeneity and outliers. The MR-Egger intercept was used to evaluate whether the results were influenced by directional pleiotropy. For metabolites with evidence to support significant causal associations, we performed reverse MR analyses to examine whether the associations may be bidirectional.

MR analyses were conducted in R using the TwoSampleMR v0.6.8 package with an FDR threshold of 5% to account for multiple testing.

### Interaction Analyses

We also examined whether there was significant evidence of interaction of the associations with vascular risk factors. Further details are in the eMethods.

### Standard Protocol Approvals, Registrations, and Patient Consents

DL1 was approved by the Multi-Centre Research Ethics Committee (04/MRE00/36). DL2 received approval from the East of England–Cambridge Central Research Ethics Committee (16/EE/0201). All participants provided informed consent.

### Data Availability

Investigators may request access to anonymized individual patient data after recruitment to DL2 has completed. Data will be shared with qualified investigators whose proposal has been approved by an independent review committee.

## Results

### Participant Characteristics

**Figure 1:**
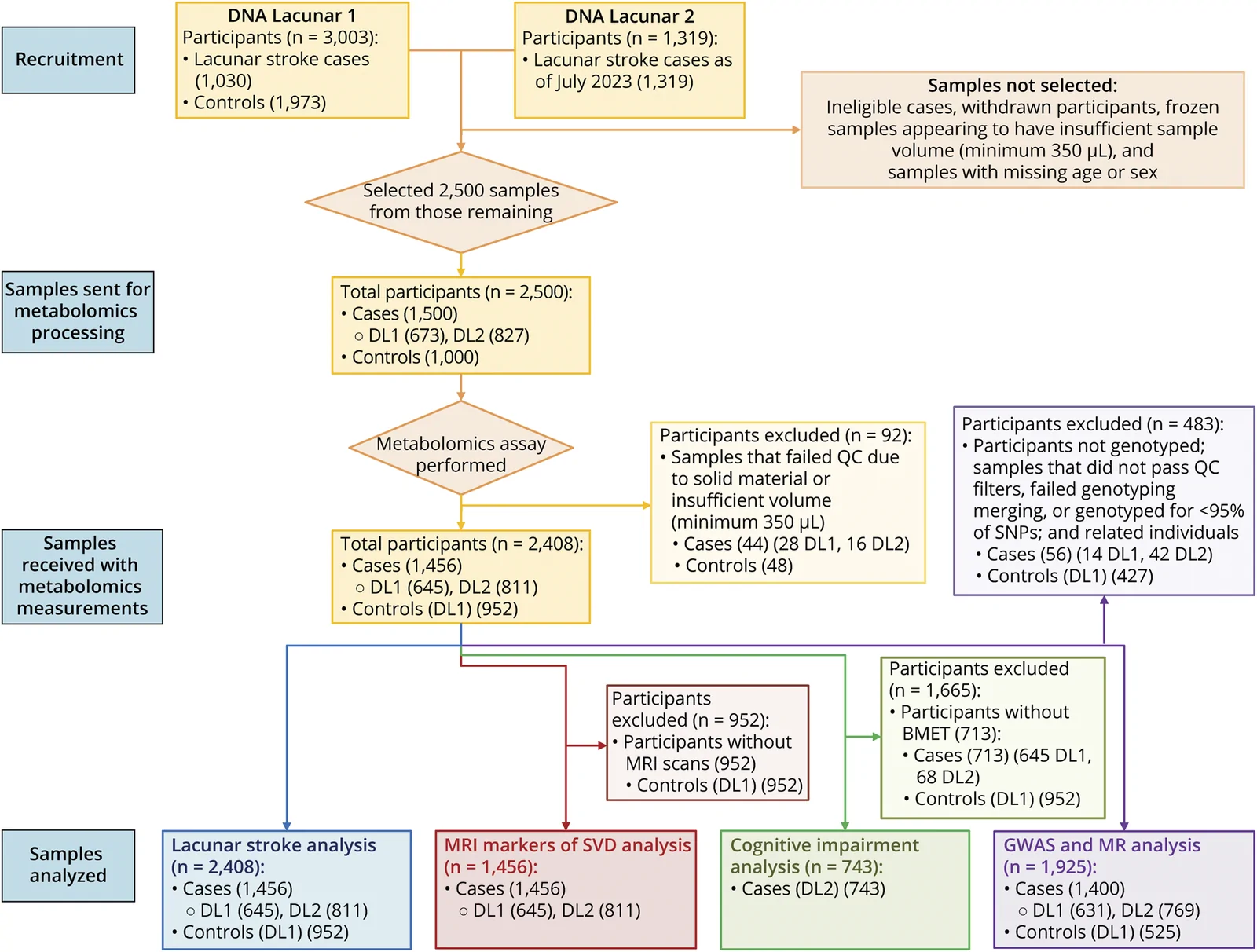
Flow Diagram of Sample Selection Process BMET = Brief Memory and Executive Test; DL1 = DNA Lacunar 1; DL2 = DNA Lacunar 2; GWAS = genome-wide association study; MR = Mendelian randomization; QC = quality control; SNPs = single-nucleotide polymorphisms; SVD = small vessel disease.

Metabolomics measurements that passed quality control were returned for 2,408 participants (DL1: 645 cases, 952 controls; DL2: 811 cases) (Figure 1; Tables 1–3). DL1 and DL2 cases were pooled. Comparison of the analyzed participants with those not selected for analysis showed no significant differences for nearly all characteristics, suggesting selection bias was unlikely (eTable 2).

**Table 1:** Demographics, Vascular Risk Factors, and Medications in Lacunar Stroke Cases and Controls

Characteristic	Total n	Cases (n = 1,456)	Controls (n = 952)	*p* Value
Age at recruitment (y)	2,408	61.92 (11.54)	57.49 (6.25)	<0.001
Male sex	2,408	962 (66.1%)	595 (62.5%)	0.0803
Hypertension	2,408	1,046 (71.8%)	443 (46.5%)	<0.001
Hyperlipidemia	2,395	955 (65.7%)	479 (50.9%)	<0.001
Type 2 diabetes	2,408	283 (19.4%)	62 (6.5%)	<0.001
Current smoker	2,407	419 (28.8%)	131 (13.8%)	<0.001
Alcohol consumption	2,395	966 (66.5%)	749 (79.5%)	<0.001
Body mass index (kg/m^2^)	2,384	28.45 (6.09)	27.38 (5.26)	<0.001
Antihypertensives	2,408	643 (44.2%)	213 (22.4%)	<0.001
Diabetic drugs	2,408	234 (16.1%)	43 (4.5%)	<0.001
Lipid-lowering drugs	2,408	438 (30.1%)	162 (17.0%)	<0.001
Antiplatelets	2,398	329 (22.6%)	110 (11.7%)	<0.001
Anticoagulants	2,397	37 (2.5%)	14 (1.5%)	0.1082

Participant characteristics are shown for lacunar stroke cases and controls from DNA Lacunar 1 and 2 with metabolomics data. Medication usage reflects treatments prescribed before the index stroke event (cases) or before recruitment (controls). Mean and SD are shown for continuous variables, while frequency and percentage are shown for categorical variables. *p* values are from *t* tests for continuous variables and from chi-squared tests for categorical variables, showing a statistical comparison of cases vs controls.

**Table 2:** Demographics, Vascular Risk Factors, and Medications in Lacunar Stroke Subtypes (ILI and MLI/LA) and Comparison With Controls

Characteristic	Total n (ILI/MLI/LA)	ILI (n = 610)	*p* Value	MLI/LA (n = 842)	*p* Value
Age at recruitment (y)	610/842	58.35 (10.86)	0.0747	64.50 (11.35)	<0.001
Male sex	610/842	397 (65.1%)	0.3269	563 (66.9%)	0.0602
Hypertension	610/842	386 (63.3%)	<0.001	657 (78.0%)	<0.001
Hyperlipidemia	610/840	391 (64.1%)	<0.001	561 (66.8%)	<0.001
Type 2 diabetes	610/842	96 (15.7%)	<0.001	186 (22.1%)	<0.001
Current smoker	610/841	179 (29.3%)	<0.001	238 (28.3%)	<0.001
Alcohol consumption	609/840	438 (71.9%)	0.0007	525 (62.5%)	<0.001
Body mass index (kg/m^2^)	600/832	28.83 (6.28)	<0.001	28.18 (5.95)	0.0028
Antihypertensives	610/842	206 (33.8%)	<0.001	436 (51.8%)	<0.001
Diabetic drugs	610/842	77 (12.6%)	<0.001	156 (18.5%)	<0.001
Lipid-lowering drugs	610/842	145 (23.8%)	0.0013	292 (34.7%)	<0.001
Antiplatelets	610/841	87 (14.3%)	0.1545	241 (28.7%)	<0.001
Anticoagulants	610/841	6 (1.0%)	0.5306	30 (3.6%)	0.0075

Abbreviations: ILI = isolated lacunar infarcts; LA = leukoaraiosis; MLI = multiple lacunar infarcts.

Participant characteristics are shown for lacunar stroke cases according to subtypes (ILI and MLI/LA) from DNA Lacunar 1 and 2 with metabolomics data. Medication usage reflects treatments prescribed before the index stroke event (cases) or before recruitment (controls). Mean and SD are shown for continuous variables, while frequency and percentage are shown for categorical variables. *p* values are from *t* tests for continuous variables and from chi-squared tests for categorical variables, showing a statistical comparison of ILI vs controls and MLI/LA vs controls.

**Table 3:** Neuroimaging Markers and Cognition in Lacunar Stroke Cases and Subtypes (ILI and MLI/LA)

Characteristic	Total n (cases/ILI/MLI/LA)	Cases (n = 1,456)	ILI (n = 610)	MLI/LA (n = 842)
WMH (Fazekas score ≥2)	1,455/610/842	591 (40.6%)	4 (0.7%)	587 (69.7%)
Presence of CMBs	718/267/451	214 (29.8%)	26 (9.7%)	188 (41.7%)
BMET total score (VCI)	743/284/459	13.13 (3.35)	13.95 (2.59)	12.62 (3.65)
BMET O/M subdomain	746/283/463	6.55 (1.88)	6.92 (1.58)	6.32 (2.02)
BMET EF/PS subdomain	735/283/452	6.67 (1.98)	7.06 (1.62)	6.43 (2.14)

Abbreviations: BMET = Brief Memory and Executive Test; CMBs = cerebral microbleeds; EF/PS = executive functioning/processing speed; ILI = isolated lacunar infarcts; LA = leukoaraiosis; MLI = multiple lacunar infarcts; O/M = orientation/memory; VCI = vascular cognitive impairment; WMH = white matter hyperintensities.

Participant characteristics are shown for lacunar stroke cases overall and according to subtypes (ILI and MLI/LA) from DNA Lacunar 1 and 2 with metabolomics data. Mean and SD are shown for continuous variables, while frequency and percentage are shown for categorical variables. BMET total score ranges from 0 to 16 where a score ≤13 is suggestive of VCI. The BMET O/M and EF/PS subdomains each range from 0 to 8 where a lower score indicates worse cognition. The direction was reversed in regression analyses so that a higher value on the subdomains corresponds to worse cognition.

Lacunar stroke cases were slightly older than controls (Table 1). Cases were more likely to have hypertension, hyperlipidemia, type 2 diabetes, and slightly higher average BMI than controls, and were more likely to smoke but less likely to consume alcohol.

When comparing lacunar stroke subtypes, ILI and MLI/LA cases had similar participant characteristics relative to controls (Table 2). However, MLI/LA cases were slightly older than ILI cases and had a higher proportion of hypertension and type 2 diabetes and a lower rate of alcohol consumption.

Cerebral microbleeds were more common in MLI/LA cases than in ILI cases, and MLI/LA cases also exhibited worse cognitive performance, as indicated by a lower average BMET total score and poorer average scores on the O/M and EF/PS subdomains (Table 3).

### Observational Associations With Lacunar Stroke

**Figure 2:**
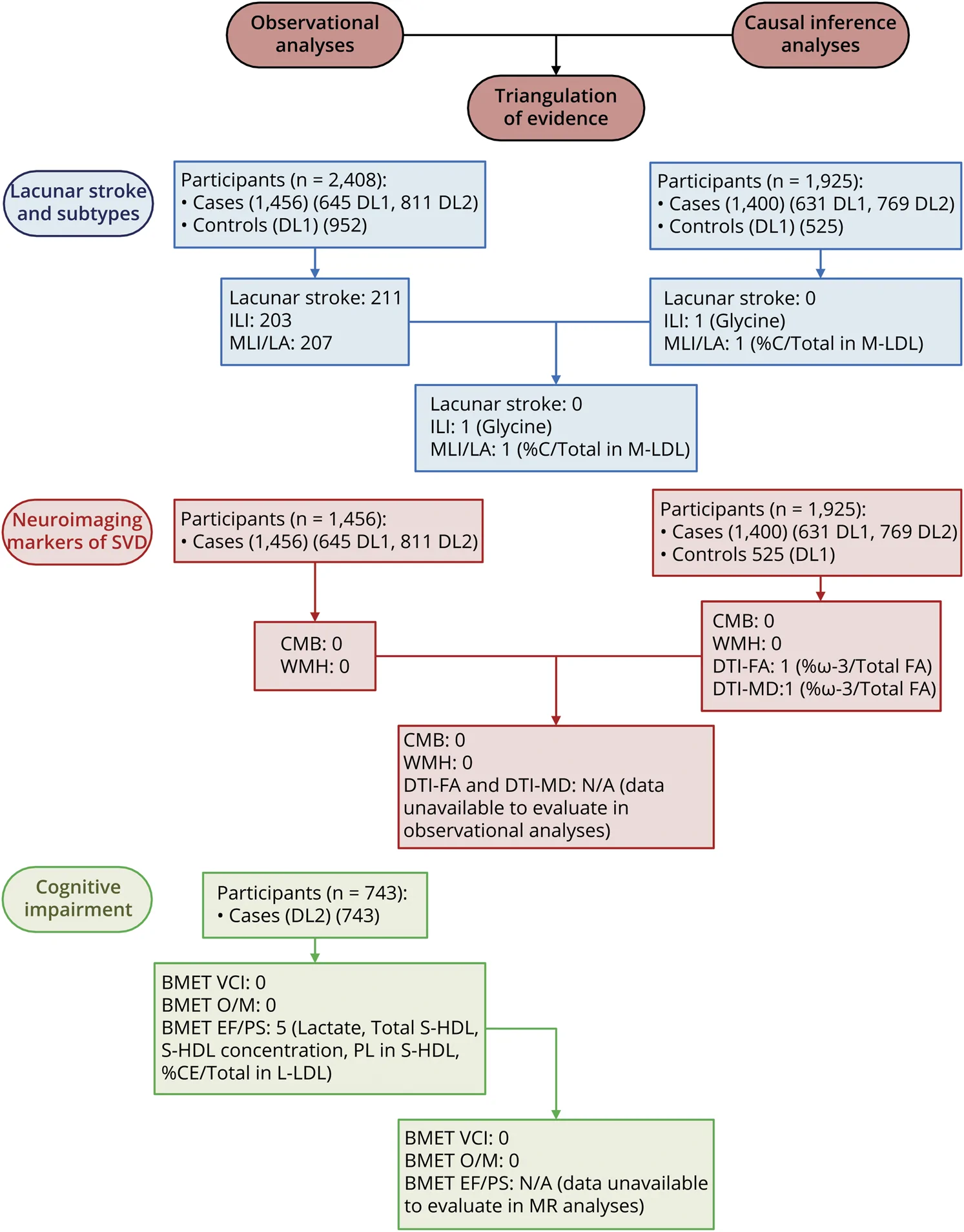
Overview of Results For 3 outcomes (lacunar stroke and subtypes in blue, neuroimaging markers of SVD in red, and cognitive impairment in green), the number of participants included in the observational and causal inference analyses is shown in the upper boxes, and the number of metabolites associated with each exposure is shown in the lower boxes. The number of metabolites with consistent associations with each exposure in both the observational and causal inference analyses is shown in the middle box under “triangulation of evidence.” BMET = Brief Memory and Executive Test; CMB = cerebral microbleeds; DL1 = DNA Lacunar 1; DL2 = DNA Lacunar 2; DTI-FA = diffusion tensor imaging fractional anisotropy; DTI-MD = diffusion tensor imaging mean diffusivity; EF/PS = executive functioning/processing speed; FA = fatty acid; ILI = isolated lacunar infarcts; LA = leukoaraiosis; L-LDL = large low-density lipoprotein; M-LDL = medium low-density lipoprotein; MLI = multiple lacunar infarcts; MR = Mendelian randomization; N/A = not applicable; O/M = orientation/memory; S-HDL = small high-density lipoprotein; SVD = small vessel disease; VCI = vascular cognitive impairment; WMH = white matter hyperintensities.

**Figure 3:**
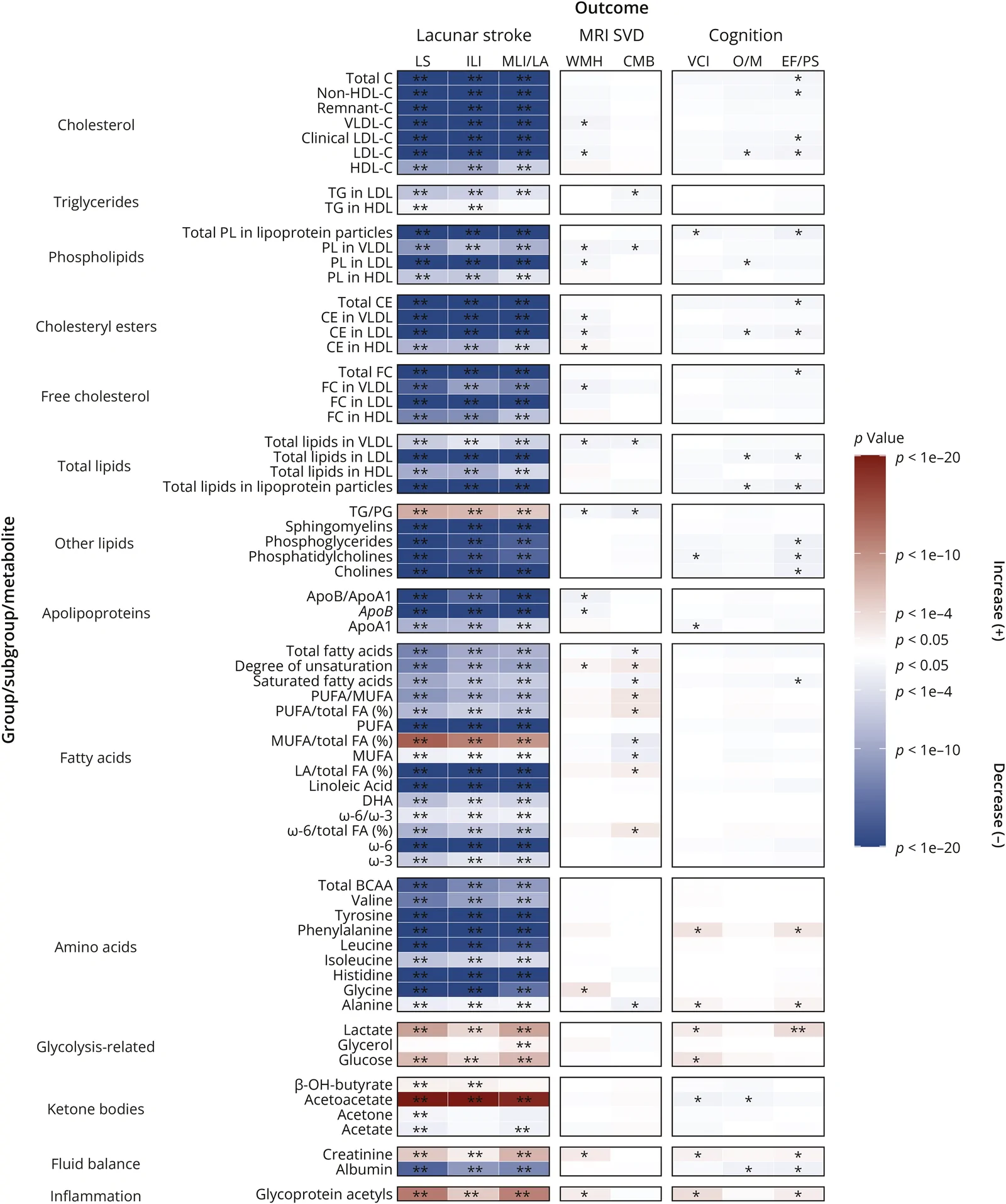
Heat Map Showing the Results of Cross-Sectional Univariate Analyses for the Association Between Metabolic Biomarkers and Lacunar Stroke, MRI Markers, and Cognitive Impairment Note: Associations of lipoprotein subclasses and relative lipoprotein lipid concentrations with lacunar stroke, MRI markers, and cognitive impairment are shown in eFigure 5. Beta estimates and *p* values were obtained from logistic or linear regression models adjusted for age and sex. Colors show the magnitude and direction of the *p* values for the association of metabolites with each outcome. Associations with beta coefficients >1 are represented in red, and associations with beta coefficients <1 are shown in blue. Associations significant at an unadjusted *p* < 0.05 are indicated with a single asterisk (*), and associations significant at an FDR-corrected *p* < 0.05 are indicated with 2 asterisks (**). BCAA = branched-chain amino acids; BMET = Brief Memory and Executive Test; C = cholesterol; CE = cholesteryl ester; CMB = cerebral microbleeds; DHA = docosahexaenoic acid; EF/PS = executive functioning/processing speed; FA = fatty acids; FC = free cholesterol; FDR = false discovery rate; ILI = isolated lacunar infarcts; LA = leukoaraiosis; MLI = multiple lacunar infarcts; O/M = orientation/memory; PL = phospholipid; SVD = small vessel disease; WMH = white matter hyperintensities.

An overview of the key findings from the observational and causal inference analyses is shown in Figure 2. In observational analyses, 211 metabolites of 250 (84%) were significantly associated with lacunar stroke at an FDR-corrected threshold of *p* < 0.05 (Figures 2 and 3; eFigures 1–3; eTable 3). Lower levels of several metabolites—including amino acids, ketone bodies, apolipoproteins, fatty acids, cholesterol and cholesteryl esters, and certain lipoprotein subclasses—were associated with an increased risk of lacunar stroke. By contrast, higher levels of metabolites such as acetoacetate, creatinine, fluid balance, glucose, glycoprotein acetyls (GlycA), 3-hydroxybutyrate, and some relative lipoprotein lipid concentrations were linked to increased risk. Similar associations were observed for both ILI and MLI/LA subtypes compared with controls (eFigures 4–5; eTable 3).

In sensitivity analyses adjusted for additional vascular risk factors and potential confounders, 211 metabolites remained significantly associated with lacunar stroke after FDR correction (eTable 4), with similar results to the basic adjustment analyses. In a sensitivity analysis further adjusted for pre-recruitment usage of medications commonly taken for stroke (e.g., antihypertensives, antiplatelets, and anticoagulants), 203 metabolites were significantly associated with lacunar stroke after FDR correction (eTable 5), overlapping substantially with the basic adjustment results.

We also conducted a PCA on 250 metabolite concentrations. A scree plot showed that the first 22 PCs explained >95% of the variance (eFigure 6A). A scatter plot of matrix loadings showed variability in metabolite levels by overall metabolite classification according to PC1 and PC2 (eFigure 6B). Fourteen of the first 22 PCs had significant associations with lacunar stroke after FDR correction (eFigure 7; eTable 6). Similar associations were observed for ILI and MLI/LA (eTable 6).

### Observational Associations With Neuroimaging Markers and Cognitive Impairment

Among the 1,456 lacunar stroke cases, we performed cross-sectional univariate analyses of metabolites with 2 MRI markers of SVD—WMH and cerebral microbleeds—adjusting for age and sex. Although 75 metabolites were nominally associated with WMH and 73 with cerebral microbleeds (uncorrected *p* < 0.05), none remained significant after correction for multiple testing (Figures 2 and 3; eFigure 1; eTable 7), indicating no robust associations with these MRI markers.

Similarly, there were no significant associations with MRI markers in sensitivity analyses adjusted for additional vascular risk factors and potential confounders (eTable 8), stroke medications (eTable 9), and duration between the stroke event and the date of blood draw (eTable 10).

Among 743 DL2 cases with cognitive testing, we assessed associations of 250 metabolites with total BMET score (VCI) and scores on the O/M and EF/PS subdomains in cross-sectional analyses adjusted for age and sex. Although no metabolites were significantly associated with VCI after correction for multiple testing (Figures 2 and 3; eFigure 1; eTable 11), 5 metabolites related to glycolysis and lipoprotein concentrations and ratios (Total S-HDL, S-HDL concentration, PL in S-HDL, %CE/Total in L-low-density lipoprotein [LDL], and lactate) showed significant associations with the EF/PS subdomain.

These 5 metabolites remained significantly associated with the EF/PS score in sensitivity analyses, including after adjustment for additional vascular risk factors and potential confounders (eTable 12), stroke medications (eTable 13), and the duration between the stroke event and the date of blood sample collection (eTable 14).

### Causal Inference Analyses for Lacunar Stroke and Neuroimaging Markers

DNA samples were genotyped in 1,958 of 2,408 participants. After excluding samples that did not pass quality control, genetic data were available for 1,925 participants (DL1: 631 cases, 525 controls; DL2: 769 cases) (eTable 15).

**Figure 4:**
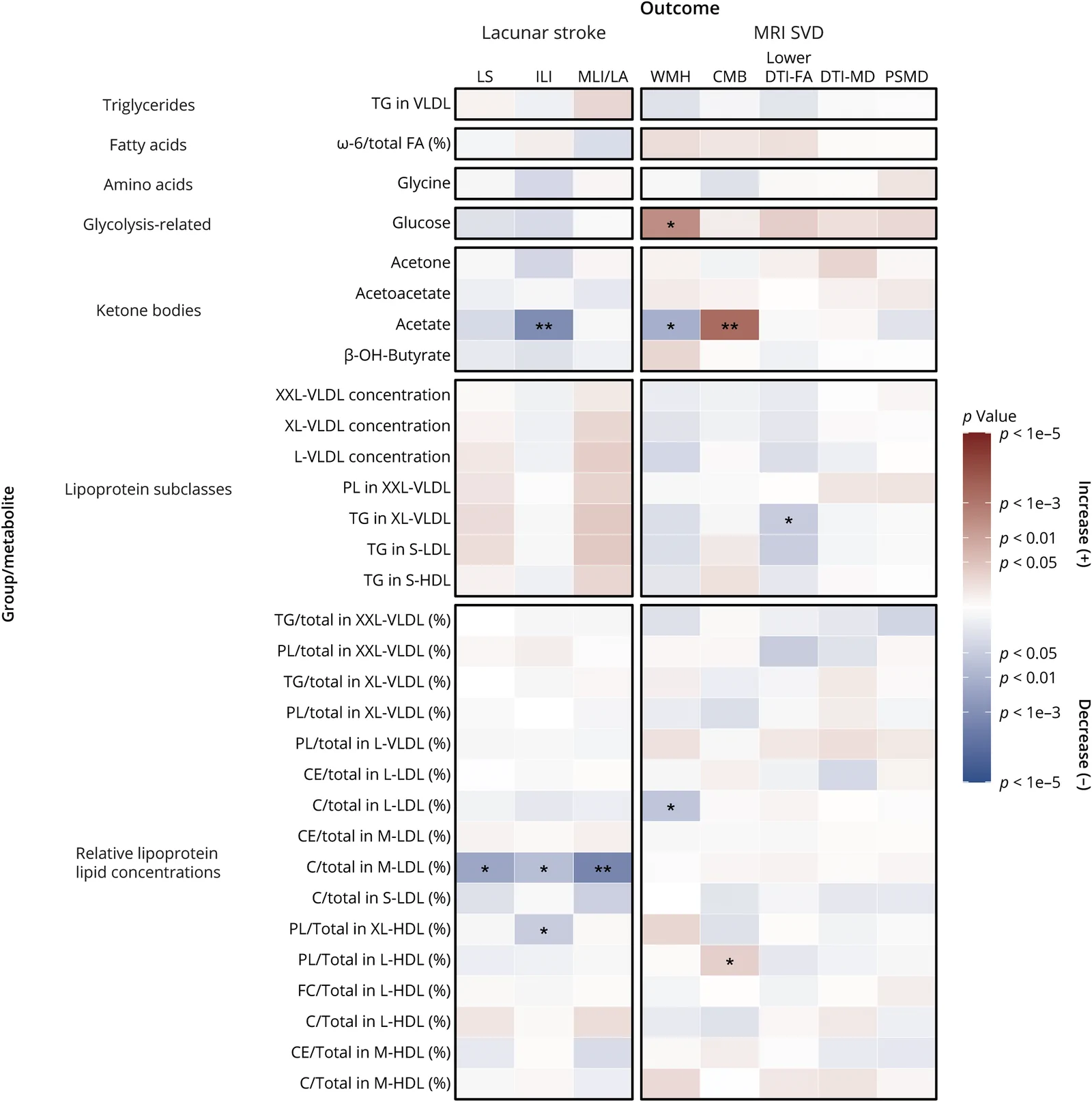
Heat Map Summarizing the Causal Estimates From Mendelian Randomization Analyses for the Association of Metabolic Biomarkers With Lacunar Stroke and MRI Markers of SVD An LD clumping threshold of *r*^2^ < 0.01 was applied. Metabolites associated with at least 1 outcome at *p* < 0.5 using the IVW method are shown. Beta estimates and *p* values were obtained from 2-sample Mendelian randomization analyses using the IVW method, or using the Wald ratio method if only a SNP was available to be used as an instrumental variable. Colors show the magnitude and direction of the *p* values for the association of metabolites with each outcome. Associations with beta coefficients >1 are represented in red, and associations with beta coefficients <1 are shown in blue. Associations significant at an unadjusted *p* < 0.05 are indicated with a single asterisk (*), and associations significant at an FDR-corrected *p* < 0.05 are indicated with 2 asterisks (**). C = cholesterol; CE = cholesteryl ester; CMB = cerebral microbleeds; DTI-FA = diffusion tensor imaging fractional anisotropy; DTI-MD = diffusion tensor imaging mean diffusivity; FA = fatty acids; FC = free cholesterol; FDR = false discovery rate; ILI = isolated lacunar infarcts; IVW = inverse-variance weighted; LA = leukoaraiosis; LS = lacunar stroke; MLI = multiple lacunar infarcts; PL = phospholipid; PSMD = peak width of skeletonized mean diff; TG = triglycerides; VLDL = very-low-density lipoprotein; SVD = small vessel disease; WMH = white matter hyperintensities.

For the 72 metabolites with ≥1 genome-wide significant association (eTable 16), we conducted 2-sample MR analyses and identified 2 metabolites significantly associated with lacunar stroke subtypes at an FDR-corrected threshold of *p* < 0.05, and 1 metabolite significantly associated with 2 neuroimaging markers of SVD (Figures 2 and 4; eTable 17). The association for glycine was only significant using a relaxed clumping threshold (*r*^2^ < 0.5) (eFigure 8; eTable 18), but the finding was validated using the generalized IVW method, which accounts for correlated instruments (eTable 19).

Genetically determined levels of glycine were associated with a reduced risk of ILI (OR: 0.704, 95% CI 0.585–0.848). Genetic predisposition to a higher proportion of cholesterol to total lipids within medium LDL (%C/Total in M-LDL) was associated with a reduced risk of MLI/LA (OR: 0.541, 95% CI 0.401–0.730). There was also suggestive evidence (uncorrected *p* < 0.05) that genetically determined levels of acetate reduce the risk of ILI and that higher %C/Total in M-LDL reduces the risk of overall lacunar stroke and ILI; however, these associations did not remain significant after FDR correction.

Genetic predisposition to higher omega-3 concentration within total fatty acids (%ω-3/Total FA) was associated with worse white matter damage on diffusion tensor imaging (DTI), indicated by lowered DTI-FA and elevated DTI-MD. There was also suggestive evidence that genetically elevated %ω-3/Total FA may also increase WMH volume, but this association was not significant after correction for multiple testing. We also found several other suggestive associations of metabolites with neuroimaging markers of SVD, namely, for genetic predisposition to a higher ratio of omega-6 to omega-3 fatty acids (ω-6/ω-3) with lowered DTI-FA and with elevated DTI-MD, genetically raised glucose levels with increased WMH volume, genetically elevated acetate with decreased WMH volume and increased cerebral microbleeds, genetically determined levels of triglycerides in very large VLDL (TG in XL-VLDL) with elevated DTI-FA, and genetic predisposition to a higher proportion of cholesterol to total lipids within large LDL (%C/Total in L-LDL) with decreased WMH volume.

The MR results were consistent in direction across sensitivity analyses (weighted median, simple and weighted mode, and MR-Egger) with no evidence of directional or horizontal pleiotropy (eFigure 9). Reverse MR analyses, in which lacunar stroke and neuroimaging markers were used as exposures and metabolite levels were used as outcomes, indicated that these associations may be bidirectional (eFigure 10; eTable 20).

### Interaction Analyses

We identified 2 metabolites (glycine and %C/Total in M-LDL) that were significantly associated with lacunar stroke subtypes in both observational and causal inference analyses. To further validate these findings, we assessed whether vascular risk factors interacted with these associations (eTable 21). Significant interactions were observed between %C/Total in M-LDL and several vascular risk factors (hypertension, smoking, BMI, hyperlipidemia, diabetes status, and age category) in relation to MLI/LA. By contrast, no significant interactions were found between glycine and vascular risk factors in relation to ILI, nor for the 5 metabolites associated with the EF/PS subdomain (Total S-HDL, S-HDL concentration, PL in S-HDL, %CE/Total in L-LDL, and lactate).

## Discussion

We conducted observational and causal inference analyses to identify metabolites associated with lacunar stroke subtypes, neuroimaging markers of SVD, and cognitive impairment. Observational analyses identified 211 metabolites associated with lacunar stroke. MR analyses identified genetic evidence consistent with causal associations of higher glycine levels with a reduced risk of ILI and higher %C/Total in M-LDL with a reduced risk of MLI/LA. Although no metabolites were significantly associated with neuroimaging markers of SVD in observational analyses, genetically elevated ω-3/Total FA was linked to 2 DTI markers of white matter damage in MR analyses. Five metabolites related to lipoproteins and glycolysis (Total S-HDL, S-HDL concentration, PL in S-HDL, %CE/Total in L-LDL, and lactate) were associated with the EF/PS subdomain, which is characteristically impaired in SVD^28^; however, we lacked genetic data to assess their causality. Overall, glycine and %C/Total in M-LDL showed the most robust and consistent associations with lacunar stroke subtypes across both observational and MR analyses.

Glycine is a simple amino acid that suppresses inflammation and reduces the risk of cardiovascular disease and dementia.^29,30^ Glycine was inversely associated with ILI in both observational and MR analyses, which has not been previously reported. There was no evidence of interaction with vascular risk factors; however, reverse MR analyses indicated bidirectional associations. The association was only significant using a relaxed clumping threshold, but this finding was validated using a generalized IVW method, so glycine might serve as a valuable diagnostic or prognostic biomarker. Circulating glycine levels can be increased through intake of collagen-rich foods and protein (e.g., bone broth, meat, fish, and legumes) or glycine powder supplementation.^31^ However, interventional studies are needed to evaluate the effect of increased glycine intake on SVD progression.

We found strong evidence for the association of higher %C/Total in M-LDL with a reduced risk of MLI/LA in both observational and MR analyses. A previous MR analysis found no association for absolute levels of cholesterol in medium LDL (C in M-LDL) with small vessel stroke,^32^ which our study confirmed. However, our findings highlight that the ratio of lipoprotein subfractions within medium LDL-C, rather than the absolute quantity, may have a greater impact on lacunar stroke risk. Another MR study found a suggestive but not statistically significant association of %C/Total in M-LDL with small vessel stroke,^33^ but our study detected a stronger, statistically significant association, which may be because MRI-confirmed lacunar stroke has improved specificity of stroke subtyping.^19^ A further MR study found associations of several metabolites with MRI-confirmed lacunar stroke,^34^ but none that overlapped with the metabolites we reported here, which may be due to differences in the populations studied (community population vs lacunar stroke cases) and metabolomics panels used (mass spectrometry vs NMR).

Reverse MR analyses showed that the causal relationship between %C/Total in M-LDL and lacunar stroke was bidirectional, and interaction analyses suggested the presence of vertical pleiotropy. Significant interactions were observed between %C/Total in M-LDL and several vascular risk factors (hypertension, smoking, BMI, hyperlipidemia, diabetes status, and age category) in relation to lacunar stroke and MLI/LA.

Although statins reduce overall LDL-C particle count, they may not optimally shift the composition or size distribution of remaining particles, so residual stroke risk may remain.^35^ Existing drugs such as fibrates and prescription omega-3 fatty acids (e.g., fenofibrate and icosapent ethyl) alter lipid subfractions, typically shifting small, dense, lipid-poor LDL particles toward larger, more buoyant particles.^36^ In addition, diets low in refined carbohydrates or high in healthy fats (e.g., Mediterranean diet) can favorably alter LDL particle composition and size, shifting away from dense, atherogenic profiles.^37^ Our findings warrant further investigation into reducing the risk of lacunar stroke, specifically by lowering the proportion of cholesterol within medium LDL-C.

Sensitivity analyses indicated that the bidirectional effects identified in the reverse MR analyses are unlikely to be explained by pleiotropic pathways, affecting metabolite levels and stroke risk independently. The MR-Egger intercept tests for horizontal pleiotropy were not statistically significant (*p* > 0.05) in most forward and reverse MR analyses, and heterogeneity (Cochran Q) was generally low, supporting validity of the findings.

That circulating metabolites were associated with cognitive performance, but not with structural neuroimaging markers (e.g., WMH), is biologically plausible. A possible explanation is that structural lesions represent historical, accumulated damage, whereas cognitive function is a dynamic clinical state influenced by real-time neurovascular coupling and current systemic metabolic profiles. Although we did not have genetic data to evaluate the causality of the observed associations with the EF/PS subdomain, consistent associations have been identified in UK Biobank for several of the same metabolites, showing that lower levels of Total S-HDL, S-HDL concentration, and %CE/Total in L-LDL were associated with increased white matter structural damage on DTI and an increased risk of all stroke and all-cause dementia.^16^ This study adds to this knowledge by showing that these metabolites are also associated with worse EF/PS scores following a lacunar stroke.

Acetate is a short-chain fatty acid produced by gut bacteria from dietary fiber and carbohydrates, which is used by astrocytes in the brain for energy derivation.^38^ Previous studies showed that acetate is associated with an increased risk of incident dementia.^16^ Our observational analyses showed that lower acetate levels were associated with MLI/LA, and our MR analyses showed that genetically lowered levels of acetate were associated with ILI, while elevated acetate levels were associated with cerebral microbleeds, so the findings are inconclusive.

Overall, glycine and %C/Total in M-LDL were the only metabolites that showed robust, significant associations in both observational and MR analyses. Although it is difficult to disentangle the causal directions, these metabolites may warrant further investigation as possible diagnostic or prognostic biomarkers. Low circulating glycine could potentially serve as a biomarker for endothelial dysfunction or early ILI. Standard clinical lipid panels typically only measure LDL-C, but our findings highlight the potential clinical utility of advanced lipid testing (e.g., NMR spectroscopy) to assess LDL particle composition and subfractions. Lower %C/Total in M-LDL might identify patients at a high risk for progressive SVD (MLI/LA) even if they have normal overall LDL-C. Future longitudinal and interventional studies are required to test whether modulating these specific metabolites translates to reduced SVD burden.

This study has several strengths. It was conducted in a unique population with MRI-confirmed lacunar stroke. Our triangulation approach combined findings from observational analyses with those from genetic causal inference analyses with numerous sensitivity analyses, which increased confidence in the robustness of the findings. We identified 2 metabolites associated with lacunar stroke subtypes, highlighting the importance of metabolic pathways in the disease pathophysiology, and indicating areas for further research in risk prediction and screening for potential therapeutic targets.

This study also has limitations. First, because blood samples in cases occurred at variable intervals after stroke onset, some observed metabolite differences may reflect acute/subacute-phase physiology rather than chronic SVD-related biology. Second, neuroimaging markers and cognitive scores were only available in a subset of DL2 cases and not assessed in controls. Restricting our analyses to poststroke patients may have introduced collider (index event) bias^39^; additionally, due to the smaller sample size, these analyses had less power to detect associations. Third, since neuroimaging and cognitive testing were not performed in controls, the absence of SVD cannot be definitively ruled out. Fourth, WMHs were not segmented and quantified but instead assessed using the semiquantitative Fazekas score, which is less sensitive to subtle differences compared with volumetric methods,^40^ potentially reducing measurement precision. Fifth, details of cerebral microbleed location (lobar vs deep) were unavailable. Sixth, cognition was assessed using the BMET, which has been previously validated to detect VCI in SVD with high sensitivity,^23^ but the test was primarily designed as a VCI screening tool and may not provide a comprehensive neuropsychological assessment. Seventh, we applied an FDR correction for multiple testing to reduce the likelihood of identifying false-positive associations, which may have inadvertently excluded some biologically and clinically meaningful associations that did not reach the threshold for statistical significance. Eighth, although we adjusted for stroke medications (e.g., antiplatelets and anticoagulants), some residual confounding may remain. Ninth, the observational analyses were cross-sectional because follow-up data were not available for clinical endpoints, so we could not evaluate associations with risk of incident cerebrovascular and neurologic conditions. We also lacked repeated metabolite measures from multiple timepoints to enable characterization of metabolite changes over the preclinical, acute, and recovery phases of SVD. Tenth, we lacked sufficient power to perform a metabolomics pathway analysis. Eleventh, significant genetic associations were only available for a quarter of the metabolites, possibly due to the small genome-wide association study (GWAS) sample size, which limited the number of metabolites that could be included in MR analyses. Twelfth, there was a partial sample overlap between our metabolite GWAS and the outcome GWAS for lacunar stroke, which could potentially bias 2-sample MR estimates due to weak instrument bias and winner's curse.^41^ Finally, our analyses were conducted in specific UK-based clinical cohorts of MRI-confirmed lacunar stroke cases and controls; further studies are required to validate these findings in other patient populations and ancestries.

In this study, we identified significant associations of 2 metabolites, glycine and %C/Total in M-LDL, with 2 lacunar stroke subtypes, with concordant associations in both observational and MR analyses. Although further research is needed to validate the findings, these results may inform future research on SVD pathogenesis and biomarker development.

## Glossary

BMET: Brief Memory and Executive Test
BMIm: body mass index
DL1: DNA Lacunar 1
DL2: DNA Lacunar 2
DTI-MD: diffusion tensor mean diffusivity anisotropy
DTIFA: diffusion tensor imaging fractional anisotropy
DWI: diffusionweighted imaging
EF/PS: executive functioning/processing speed
FDR: false discovery rate
FLAIR: fluid-attenuated inversion recovery
GWAS: genome-wide association study
HDL: high-density lipoprotein
ILI: isolated lacunar infarcts
IVW: inverse-variance weighted
LA: leukoaraiosis
LDL: low-density lipoprotein
MLI: multiple lacunar infarcts
MR: Mendelian randomization
O/M: orientation/memory
PC: principal components
PCA: principal component analysis
PL: phospholipid
SVD: small vessel disease
VCI: vascular cognitive impairment
WMH: white matter hyperintensities
